# Incidence of Flexor Tendon Injuries in Complex Intra-Articular Distal Radius Fractures Fixed With Volar Rim Plate Osteosynthesis

**DOI:** 10.7759/cureus.29852

**Published:** 2022-10-03

**Authors:** Wei Siong Chua, Sallehuddin Hassan, Anizar Faizi Anoar

**Affiliations:** 1 Orthopaedics and Traumatology, Hospital Kuala Lumpur, Kuala Lumpur, MYS; 2 Orthopaedics, Hospital Sultanah Bahiyah, Alor Setar, MYS

**Keywords:** malay dash questionnaire, malaysia, flexor tendon rupture, flexor tendon irritation, distal radius, volar rim

## Abstract

Aim: The volar rim plate is anatomically contoured to provide buttressing of distal radius fragments including the lunate fossa. The low-profile design of the plate minimizes flexor tendon irritation. This study aims to determine the Disabilities of the Arm, Shoulder, and Hand (DASH) score and the presence of flexor tendon irritation at around one-year post operation.

Method: Between June 2020 and May 2021, all patients with AO-23B3 and AO-23C (1-3) distal radius fractures who were treated with a volar rim plate fixation were included in this study. At 12 months after surgery, the patients were evaluated utilizing DASH score as a routine as well as evidence of flexor tendon rupture or irritation.

Results: Twenty-five patients were finally included in this study. Of these, three required additional dorsal plating for dorsal subluxation, four required fixation of ulna styloid with tension band wiring, and the rest (18) had volar rim plate fixation in isolation. The mean DASH score was 16.3. Two of the patients had flexor tendon irritations; one in the middle finger and another in the ring and little finger. None had flexor tendon rupture.

Conclusion: The volar rim plate is designed to tackle complex intra-articular distal fractures which are near the watershed line. There was no evidence of flexor tendon irritation on routine follow-up. The outcome was satisfactory in this small series despite the complexity of the fractures. Evidence of flexor tendon irritation requires prompt attention to enable early implant removal.

## Introduction

Distal radius fractures have different outcomes depending on their severity. Intra-articular fractures, especially those that involve the lunate facet, usually fare poorly with conservative management due to inadvertent carpus subluxation [1-4]. Due to the presence of lunate volar rim projection [3,5], normal conventional locking plates are unable to address this fragment without violating the radiocarpal joint via unavoidable screw trajectory [1,2,6,7]. In addition to that, if the volar marginal rim fragment is not addressed, it will be absorbed due to its avascularity [4]. Moreover, if the lunate fossa fragment is below 15 mm, a potential loss of reduction can happen if treated with conventional volar locking plates [3].

Methods have been devised to address these issues like wire loop fixation, fragment-specific implants, bridge plating, spanning external fixators, K-wires, and compression screws [1-3,7,8]. Recently, a pre-contoured plate has been designed to address the different fracture fragments. The volar rim plate enables placement distal to the watershed line and beyond the distal volar lunate projection [3,9]. However, this implant is not without concerns as flexor tendon injuries are reportedly as high as 12% following volar rim osteosynthesis [9].

In our review, we aim to evaluate the evidence of flexor tendon disturbance following volar rim plate fixation and their respective Disabilities of the Arm, Shoulder, and Hand (DASH) score of the affected upper limb at least six months following surgery.

## Materials and methods

Between July 2020-May 2021, databases were extracted from operation theatres and implant vendors at Hospital Sultanah Bahiyah, Alor Setar. Only patients with volar rim plate fixation following trauma are included. Of these patients, only those who successfully completed mDASH during routine follow-up at six months were included. The original DASH outcome comprises 30 questions designed to measure physical function with any upper limb musculoskeletal disorders. In our study, we use mDASH, which is the Malay language validated version of the DASH score by Al-Husuny et al [10]. Indications for the use of volar rim plates were explored from our databases. Radiographs of the fracture were then obtained and classified according to AO by a single-hand surgeon. At six months after surgery, the radiographs were evaluated for any loss of reduction and carpus subluxation. Records were then perused for the past year for evidence of flexor synovitis: crepitus while moving, loss of full active digit flexion, or pain during digit flexion. The scores and averages were documented. The local ethics committee did not request an institutional review board (IRB) since this is a retrospective study without identifying information.

Surgical method

The incision is usually made parallel and radial to flexor carpi radialis (FCR) tendon. Upon retracting the flexor pollicis longus (FPL) tendon to the ulnar side, the pronator quadratus is revealed. The white fibrous transitional zone overlying the volar rim is identified. An L-shaped incision is made over this transitional fibrous zone and lateral edge of the pronator quadratus, revealing the underlying distal radius. Fracture is reduced and held with K-wires followed by the placement of the volar rim plate firmly placed on the distal radius. Fluoroscopy was performed to ensure screw purchase accounted for all fracture fragments without violating the radiocarpal joint/sigmoid notch. Pronator quadratus is routinely repaired to protect the volar rim plate whenever possible.

## Results

Within the time frame of the study, there were 80 patients who had volar rim plate fixation for distal radius fractures, Of these, there were 25 patients who had filled the mDASH questionnaire to date and were included in this study. The age range was 21-67 years old with an average of 38.9. Twenty of them were males, while five were female (Table 1). Out of the 25, 20 had isolated fixation of the distal radius with volar rim plate, three had additional fixation of ulna styloid, and two required complementary fixation of the dorsal fragment via buttress plate. Fracture fixation stability, as well as intra-articular rim congruity, was checked with fluoroscopy intraoperatively through a full range of wrist movements. Post-operative radiographs show a reduction of carpus subluxation and fracture reduction immediately and on follow-up.

**Table 1:** Summary of 25 patients in this study AO: AO classification, DASH: Disabilities of the Arm, Shoulder, and Hand, mDASH: Malay language validated DASH, F/U: follow-up

	GENDER	AGE	AO	VOLAR RIM FIXATION INDICATIONS			COMPLICATIONS	mDASH	F/U
									(MTHS)
Pt 1	M	26	c3	lunate fragment			none	68.3	12
Pt 2	M	21	c2	lunate fragment	<15mm fragment		none	10.8	11
Pt 3	M	41	c3			carpus subluxation	none	6.7	13
Pt 4	F	52	c2	lunate fragment	<15mm fragment		none	10.8	10
Pt 5	M	41	c3		<15mm fragment	carpus subluxation	none	2.5	11
Pt 6	M	32	c3		<15mm fragment		none	9.2	13
Pt 7	M	45	c3		<15mm fragment	carpus subluxation	none	22.5	11
Pt 8	M	36	c3		<15mm fragment		none	25.8	15
Pt 9	M	41	c2		<15mm fragment		none	15.0	12
Pt 10	M	24	c3			carpus subluxation	none	14.2	15
Pt 11	M	33	c3		<15mm fragment	carpus subluxation	none	11.7	14
Pt 12	F	39	c2		<15mm fragment		none	9.2	13
Pt 13	F	51	c3		<15mm fragment		none	3.3	10
Pt 14	M	62	c3		<15mm fragment		none	16.7	11
Pt 15	M	35	c3			carpus subluxation	none	20.8	12
Pt 16	M	33	c3		<15mm fragment	carpus subluxation	none	54.2	11
Pt 17	M	31	c3	lunate fragment			none	0.0	12
Pt 18	M	56	c3		<15mm fragment		none	0.0	12
Pt 19	M	32	c3	lunate fragment			ring/little finger flexion irritation	44.2	11
Pt 20	F	57	b3		<15mm fragment	carpus subluxation	middle finger flexion irritation	11.7	8
Pt 21	F	36	c3		<15mm fragment		none	19.2	13
Pt 22	M	25	c3		<15mm fragment	carpus subluxation	none	1.7	9
Pt 23	M	28	c1		<15mm fragment		none	3.3	13
Pt 24	M	28	c3		<15mm fragment		none	12.5	8
Pt 25	M	67	c2		<15mm fragment		none	15.0	11
AVG		38.9						16.4	11.6

**Figure 1:**
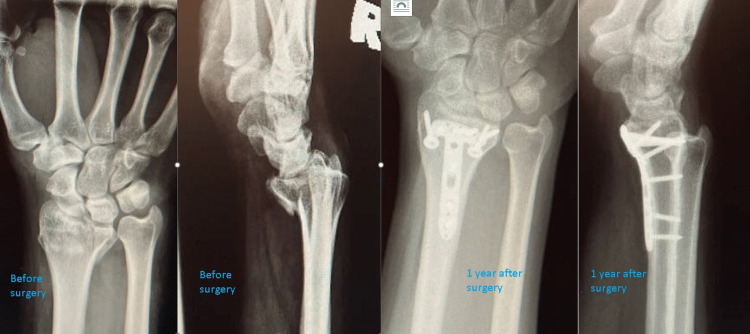
An example of AO 23C3 distal radius fracture treated with volar rim fixation by addressing the lunate fossa fragment

Among our 25 patients, 18 of them has AO classification C3, five with C2, and one of C1 and B3 each. The indications of fixation using the volar rim plate include the presence of a lunate fragment (five patients), and a fracture fragment less than 15 mm (19 patients) from the volar rim (Figure 1). There were nine patients with carpus subluxation. Fifty-five other patients who had volar rim plate fixation were unable to meet the 12-month routine follow up due to the COVID pandemic. Assessments clinically include the presence of pain during arc of motion of fingers as well as loss of active motion [1]. The mean mDASH score was 16.4. Of the 25 respondents, two people had flexor tendon irritation; one with middle finger irritation, and another with ring and little finger irritation. None had evidence of flexor tendon ruptures as they were all able to perform full finger flexion actively.

## Discussion

Fractures of the distal radius, particularly those that involve the volar rim and the small lunate fragments, are prone to carpus subluxation if not addressed properly. CT scan shows there is a small projection of the lunate facet from the radius flat surface, with a mean thickness of 5 mm and 19 mm width [3]. The fixation of this fragment poses a great challenge without the correct implants. This led to the choice of a volar rim implant to address this issue in our population.

The volar rim plate is specifically designed to address fragments that extend to the watershed line. This line is defined as a ridge that is found within 2 mm of the ulna side of the joint line and 10-15 mm within the radial side [9]. This low-profile plate addresses fractures of the AO23B3 and AO23C3 well by securing the different fragments with good reduction of the distal fracture fragments, thereby facilitating the reduction of carpus subluxations. Moreover, this implant can address dorsal fragments from a single volar incision [3]. However, in our practice, we found that certain dorsal fragments, in particular the dorsal ulnar fragment, were difficult to stabilize via the volar approach and needed to be supplemented with the dorsal plate. Hence, the attending physician must cater their surgical plans accordingly.

Numerous complications following distal radius fixation have been documented. Among them are nerve irritation, post-traumatic arthritis, loss of reduction, scapholunate dissociation, and flexor tendon disturbances [3,6]. Flexor tendon disturbances encompass a wide array of problems that range from flexor tenosynovitis to irritation and eventual rupture. The onset of symptoms of flexor tendon rupture can happen as early as three months or as late as five years [11]. As the volar rim plate is designed to sit on the volar rim, this is believed to cause flexor tendon problems, particularly to the FPL, because the rim is the most prominent part of the distal radius. The distance between the FPL tendon and volar rim plate is found to be 1.3 mm in an anatomic study [5]. Despite this, some reports suggest that flexor tendon ruptures are more likely due to improper implant placement, loss of reduction, or malreduction [5,6,12]. Two of our patients with flexor tendon irritation are scheduled for early implant removal.

Several literature studies did not have any reported cases of tendon ruptures among their distal radius fixations [13,14]. However, when compared with two different implants, Soong et al. found there were three ruptures out of 75 fixations on an average of 20 months after surgery in a specific implant group [9]. Kitay et al. reported eight flexor tendon ruptures; however, they did not state the proportion of ruptures from total distal fixations [12]. Our findings of no flexor tendon ruptures are consistent with Marcano et al. and Naito et al. [13,14].

Kitay et al. report that the eight cases of tendon rupture happen at least six months after surgery with a mean of 3.8 years [12]. Hence they recommend routine removal of the implant at six months. On the contrary, Spiteri et al. recommend a review at six months to review symptoms of flexor tendon irritation to aid the possible decision of implant removal [6]. During routine follow-up, clinical tests to determine flexor tendon irritation include cracking or pain during thumb mobilization, localized swelling, and weakness/loss of digit flexion [5,8]. Any positive finding suggestive of flexor tendon irritation should prompt the attending physician to consider early removal of the implant and perform a routine examination of the flexor compartment tendons intraoperatively in the same setting. Catering to our local setting, a review at six months to look for flexor tendon irritation symptoms is a reasonable protocol, to plan for early removal of the implant. Other factors that are worth looking into include the design of the plate, screw head prominence, and the locking system. Our DASH score average is 16.4, which is consistent with Spiteri et al. [6]. This is slightly higher than other average scores in different literature [15-17]. This might be attributed to the complexity of the initial fracture. Looking at the wide range of scores ranging from 0-68.3, there should be different preinjury scores of the patients. Moreover, the DASH may be affected by concurrent ailments in the same upper limb that is not related to the distal radius. Due to the limitation of the retrospective design of these data, we did not have a DASH score before the trauma for comparison. Another profound limitation in this study was the inconsistent documentation of the pronator quadratus repair. This did not allow us to correlate the link between pronator repair and flexor tendon irritation.

## Conclusions

The volar rim remains a versatile implant to address fragments of distal radius which is otherwise unattainable with conventional distal radius locking plates. This implant is safe for distal radius fractures as long as patients are routinely checked for flexor tendon irritation symptoms post operatively.
